# Current Utility of Plant Growth-Promoting Rhizobacteria as Biological Control Agents towards Plant-Parasitic Nematodes

**DOI:** 10.3390/plants9091167

**Published:** 2020-09-09

**Authors:** Pratima Subedi, Kaitlin Gattoni, Wenshan Liu, Kathy S. Lawrence, Sang-Wook Park

**Affiliations:** Department of Entomology and Plant Pathology, Auburn University, Auburn, AL 36849, USA; pzs0066@auburn.edu (P.S.); Kmg0073@tigermail.auburn.edu (K.G.); wzl0028@auburn.edu (W.L.)

**Keywords:** direct/indirect antagonism, induced systemic resistance (ISR), jasmonic acid, management, salicylic acid, systemic acquired resistance (SAR)

## Abstract

Plant-parasitic nematodes (PPN) are among the most economically and ecologically damaging pests, causing severe losses of crop production worldwide. Chemical-based nematicides have been widely used, but these may have adverse effects on human health and the environment. Hence, biological control agents (BCAs) have become an alternative option for controlling PPN, since they are environmentally friendly and cost effective. Lately, a major effort has been made to evaluate the potential of a commercial grade strain of plant growth-promoting rhizobacteria (PGPR) as BCAs, because emerging evidence has shown that PGPR can reduce PPN in infested plants through direct and/or indirect antagonistic mechanisms. Direct antagonism occurs by predation, release of antinematicidal metabolites and semiochemicals, competition for nutrients, and niche exclusion. However, the results of direct antagonism may be inconsistent due to unknown endogenous and exogenous factors that may prevent PGPR from colonizing plant’s roots. On the other hand, indirect antagonism may occur from the induced systemic resistance (ISR) that primes whole plants to better fight against various biotic and abiotic constraints, actuating faster and/or stronger defense responses (adaption), enhancing their promise as BCAs. Hence, this review will briefly revisit (i) two modes of PGPR in managing PPN, and (ii) the current working models and many benefits of ISR, in the aim of reassessing current progresses and future directions for isolating more effective BCAs and/or developing better PPN management strategy.

## 1. Introduction

Plant-parasitic nematodes (PPN) are biotrophic parasites that cause billions of dollars in economic damages on major food crops including corn (*Zea mays* L.), potato (*Solanum tuberosum* L.), soybean (*Glycine max* (L.) Merr.), as well as an important fiber crop, cotton (*Gossypium hirsutum* L.) [1]. Thus, a variety of management strategies using chemical nematicides, cultural control, resistant varieties and biological control (BCA) have long been tested, and employed to manage PPN. Nematicides, however, may not always be environmentally friendly, having hazardous and non-target impacts on flora and fauna including many natural enemies [2]. Although nematicides may initially reduce PPN populations and provide protection from their damage, these plants may allow the increase of populations at the end of the growing season. The following year will require an additional application of costly nematicides [3]. On the other hand, cultural control and resistant cultivars are not always available [4,5]. Hence, increasing attention has lately been drawn to the potential use of BCAs as an effective, inexpensive, and environmentally friendly tools of PPN management [3].

BCAs, a natural enemy or predator, can work by direct or indirect antagonism. Direct antagonism occurs in general through a predation, a release of toxic metabolites or a competition for nutrients and niche exclusion, whereas indirect antagonism can operate via induced systemic resistance (ISR) or systemic acquired resistance (SAR) [3]. ISR is defined as an enhanced plant resistance induced by plant growth-promoting rhizobacteria (PGPR) and typically employs jasmonic acid (JA) and ethylene (ET) hormone signaling [6]. SAR is also a form of induced plant resistance activated by an exposure to necrotizing biotrophs, which requires the accumulation of salicylic acid (SA) in infected and systemic tissues [7]. Over the last several decades, increasing efforts have been invested to examine the efficacy and practicality of PGPR in the hope of commercializing them as economic BCAs (Table 1, Table 2, Table 3 and Table 4). PGPR are well proven “effective biostimulants”, facilitating plant growth and productivity, and being concurrently able to improve plant resistance against a broad range of microbial pathogens and insect herbivores. Moreover, PGPR can prime plants to better adapt or acclimate towards various abiotic stresses including drought, salinity, and extreme temperatures [8]. Therefore, the successful development of commercial PGPR (BCAs) will greatly aid in upgrading plants’ own survival capacity against PPN as well as many other ecological constraints, without “trading off” yield potential. However, the current utility of PGPR in the field is still limited, largely due to our little knowledge of, in the molecular, biochemical and cellular levels, their mode of interactions with PPN and plants. Hence, this review will briefly revisit current working models of the mode of actions of PGPR with a focus of PPN management, and pinpoint information gaps within, which help revamp unique and alternative prospective for the future studies.

## 2. Recent Increases in Agronomic Burden by PPN

PPN, microscopic roundworms, belonging to the phylum Nematoda that are among the most abundant animal on earth; over 4100 species have been found in a variety of environmental conditions. The majority of PPN live in the film of moisture surrounding soil particles and plant roots. PPN have protrusible stylets or mouth spear to enter root tissues [1]. On the basis of their feeding habitats, PPN are chiefly classified into two groups, ectoparasites, and endoparasites. Ectoparasites feed from the outside of root surfaces without entering into plant roots. Thus, they could be more susceptible to environmental stresses and predators, and causing lesser damage to plant roots, than endoparasites. Endoparasites are able to penetrate completely or partly into plant roots during the infection process. This causes physical injury as well as allows secondary damages by bacteria and fungi infected along with or after PPN [113]. Damages caused by endoparasitic nematodes including *Meloidogyne* spp. (root-knot nematodes; RKN), *Heterodera* spp., *Globodera* spp. (cyst nematodes), *Pratylenchus* spp. (lesion nematodes), and *Rotylenchulus* spp. (reniform nematodes) are estimated to result in an annual loss of ~13% (~$216 billion) worldwide [114]. For instance, two genera of PPN, *Meloidogyne* spp. and *Heterodera* spp., alone cause the ~10% reduction of major food production including wheat (*Triticum aestivum* L.), rice (*Oryza sativa* L.), corn, potato, and sweet potato (*Ipomoea batatas* L.), which totaled an estimated loss of ~$80 to 114 billion per year. *Rotylenchulus* spp. has also emerged as a major threat in cash crops, cotton, and soybean, over the last decade throughout the southern regions of the US causing an estimated yield loss of over $200 million annually [1,115,116,117]. Hence, the establishment of effective and sustainable management program to control PPN has become an urgent task across the nations. However, current methods of integrated PPN management are rather restricted, when compared to that for other insect and animal pests, because PPN are microscopic and generally attack the underground portion of plants. The damage caused by PPN also are often not correctly identified because the aboveground symptoms mimic other diseases and environmental root-limiting factors delaying proper management [118,119].

### Current Management Method against PPN

The current integrated pest management (IPM) program aimed at PPN largely strategizes the combined uses of crop rotation, tolerant/resistant cultivars, and nematicides. These methods, however, are still inadequate, expensive and/or cause numerous unexpected ecological and social drawbacks [120,121]. Presently, the most common strategy to manage PPN infections and field infestations is the application of nematicides (e.g., aldicarb, fluazaindolizine, fluensulfone, fluopyram, oxamyl, and terbufos) [122,123]. Nematicides have been an effective management tool; however, chemical nematicides are harmful to the environment, dangerous to apply and very costly [118]. Therefore, crop rotation has long been recommended as an alternative and potentially effective cultural practice to reduce nematode population density [5], but is not be a feasible option for all crops and farmers. For example, in the southern areas of the US, one of the most efficient crop rotations schemes for RKN, *Meloidogyne incognita* (Kafoid and White, 1919) [124] infested fields include the nonhost crop of peanut (*Arachis hypogaea* L.) [5,125]. However, peanuts require different harvesting equipment compared to cotton, soybeans, and corn, the most prominent row crops in the region. This harvesting equipment can range into the hundreds of thousands of dollars, and therefore may not be an economically feasible strategy. 

Resistant cultivars increasingly have become available, preventing PPN from reproducing at as high a level as with susceptible cultivars; however, these cultivars are often accompanied by decreased yield [126]. Thus, some groups have advocated the use of tolerant cultivars that do not decrease the PPN reproduction but have little effect on yield potential [127]. The crucial drawback of tolerant cultivars though is that they cause an increase in the field population density of PPN. Thus, the use of resistant and tolerant cultivars is the most successful when used as a part of IPM system. Hence, alternative strategies must be looked at for effective, sustainable management of PPN. One, emerging option is biological control, which encompasses beneficial microbes such as PGPR that can reduce the impact of PPN, and other biotic attackers and abiotic stresses, as well as synergistically help the plant enhancing growth and development of various plant species [8].

## 3. Current utility of PGPR as BCAs of PPN; Mode of actions of PGPR

Recent studies have proposed that biological control could be a cost-effective approach to manage PPN when compared to the conventional methods. Especially, PGPR have emerged as promising BCA candidates [128,129]. PGPR are non-pathogenic bacteria, known to enhance plant growth and development in both non-stressed and stressed conditions by direct and indirect mechanisms [130]. The direct mechanism describes PGPR as bio-fertilizers producing organic compounds that promote plant growth or increase uptake of soil nutrients. Indirect mechanisms refer to PGPR-dependent biocontrol, including the production of antibiotics, Fe chelators (called as siderophores), and external cell wall degrading enzymes (e.g., chitinase and glucanase) that perhaps hydrolyze the pathogen (i.e., fungus) cell wall [131]. In line with this scenario, a number of PGPR, frequently from the genera of *Agrobacterium*, *Bacillus*, *Paenibacillus* and *Pseudomonas*, have been documented to reduce PPN population density through different mechanisms like parasitism, production of hydrolytic enzymes and antinematicidal metabolites (direct antagonism; Table 1), and inducing systemic resistance (indirect antagonism; Table 2) [130].

### 3.1. Direct Antagonism of PGPR against PPN

Direct antagonism of PGPR can prevent egg hatch, and/or the growth and reproduction of PPN, mainly through predation, and release of toxins or hydrolytic enzymes including hydrogen cyanide (HCN), 2,4-diacetylphloroglucinol (DAPG), chitinases, glucanases, proteases, and lipases (Table 1). The best characterized, although not necessarily PGPR, predatory bacterium is *Pasteuria penetrans* (ex Thorne, 1940) [42], which directly attacks and colonizes the body cavity of RKN (*Meloidogyne* spp.). Once attached on the cuticle surface of RKN, *P. penetrans* spores begin to form germination tubes that penetrate and rupture the cell membrane of PPN [132]. Likewise, *P. thornei* [133] can attack root-lesion nematodes (RLN, *Pratylenchus* spp.) and *P. nishizawae* [45] is able to attack the cysts nematode of two genera, *Heterodera* spp., and *Globodera* spp. [44].

Indeed, the exogenous application of *P. penetrans* demonstrated drastic decreases in the population density of *M. javanica* (Treub, 1885) [123] in sugarcane (*Saccharum officinarum* L.), by an average of 97.5% [43]. Thus, a few *Pasteuria* spp. have been made into commercial products; e.g., Clariva pn, Naviva ST and NewPro (Table 3). However, these products appear to be only active on one or two target species limiting actual practicality and use for field conditions. Producers often look for products that confer a broader spectrum of disease resistance, as well as defense responses against PPN.

On the other hand, a number of PGPR belonging to *Bacillus* spp. and *Pseudomonas* spp. produce nematicidal compounds. For example, several strains of *Pseudomonas fluorescens* (Flüge, 1886) [134] reduce potato cyst nematodes (PCN, *Globodera* spp.) and RKN (*M. incognita*) levels in vitro and/or in soil conditions by releasing DAPG, a phenolic antiphytopathogenic metabolite [49]. The wild type (WT) bacterium can reduce the motility of juvenile PCNs by 85%, comparing to 27% by mutant strains that disrupted DAPG productions [49]. On the other hand, *P. fluorescens* strain CHA0 and *P. aeruginosa* (Schröter, 1872) [135] produce the volatile compound HCN, an extremely poisonous toxin that adversely affects the proper functions and formations of mitochondria in serval RKN spp. (e.g., *M. incognita* and *M. javanica*) [46,50]. Interestingly, *P. fluorescens* strain CHA0 also releases both DAPG and pyoluteorin, two secondary metabolites that can significantly decrease population density of *M. javanica* in vitro and in a soil setting [51,53].

Several hydrolytic enzymes produced by PGPR are also able to inhibit the synthesis and maintenance of cell walls and membranes, as well as prevent the formation of cellular organelles [136]. Chitinases and discrete proteolytic enzymes produced from *Bacillus cereus* [137], *B. firmus* [138], *B. licheniformis* (Weigmann, 1898) [139], *B. megaterium* [140], and *B. subtilis* (Ehrenberg 1835) [141], as well as *Corynebacterium paurometabolu* [142] are proposed to be responsible for suppressing the multiplication of diverse RKN and cysts nematodes (see Table 1).

However, current attempts to commercialize PGPR for PPN management has had limited success [143]. A major concern has been that PGPR is inconsistent in managing PPN in the field, possibly due to many endogenous and exogenous factors limiting PGPR root colonization. Factors include other plants, variable soil conditions, and various rhizospheric metabolites and organisms [144]. Thus, there is a need to further investigate enhancing and/or optimizing the BCA activity of PGPR. Many studies now have turned into investigate the ability and a potential efficacy of indirect antagonism (i.e., ISR) of PGPR against PPN as an alternative, practical way to ensure the improvement of the defense capacity of plants against PPN, as well as other pathogens and abiotic constraints, and concurrently to enhance plant growth and development.

### 3.2. Indirect Antagonism, ISR of PGPR against PPN

Indirect antagonism of PGPR against PPN that occurs by ISR, is also referred to as PGPR-mediated priming, which systematically equips the "whole" plants to better cope with environmental constraints, actuating faster and/or stronger defense responses (adaption) to a subsequent exposure to various biotic and abiotic stresses. ISR is nonspecific in nature, and provides plants “a long-lasting protection” and “a broad-spectrum disease resistance (defense responses)” against various pathogenic microbes, insect herbivores and pests including PPNs; e.g., *M. incognita*, *M. javanica*, *Heterodera glycines* [145], *H. cajani* [146] and *Globodera pallida* [147] Behrens, 1975 (Table 2) [6]. In fact, ISR developed by various *Bacillus* spp. and *Pseudomonas* spp., including *B. subtilis*, *B. amyloliquefaciens* (ex Fukumoto, 1943) [148] and *P. fluorescens* have demonstrated promising results suppressing PPN in both dicotyledonous (*Arabidopsis*, bean (*Fabaceae* spp.), carnation (*Dianthus caryophyllus* L.), cucumber (*Cucumis sativus* L.), radish (*Raphanus sativus* L.), tobacco (*Nicotiana tabacum* L.), and tomato (*Solanum lycopersicum* L.)) as well as monocotyledonous (rice, maize, and sugarcane) plants (see Table 2).

However, molecular mechanisms underlying its occurrences are still elusive. Thus far, a majority of studies have foregrounded the potential, functional relevance of JA and ET hormones in ISR development, as mutant plants disrupted in JA/ET biosynthesis or signaling exhibited impaired PGPR-induced priming (reviewed in [6]). Moreover, exogenous application of JA could demonstrate ISR development against RKN (e.g., *M. incognita*, *M. graminicola* [149], and *M javanica*), cyst nematodes (e.g., *H. avenae* (Wollenweber, 1924) [150], *H. schachtii* [151]), and root lesion nematode (*Pratylenchus neglectus* (Rensch, 1924) [152]) in various plant species [153,154]. However, the root colonization of PGPR did not appear to stimulate the production of JA/ET in systemic (leaf) tissues, nor did they induce the expression of JA/ET-responsive genes in leaves [155,156]. Instead, ISR could potentiate JA/ET-responsive genes. When plants exposed/treated with PGPR were later challenged with other biotic stressors (e.g., microbial pathogens, insects, or herbivores), ISR facilitated a more rapid and/or enhanced level of induction of JA/ET-responsive genes, *VSP*, *PDF1.2*, and *HEL* [156,157].

On the other hand, selective results from earlier studies argued otherwise. Root inoculations of PGPR immediately triggered JA production and signaling transductions in systemic (leaf) tissues, which in turn heighted the state of defense responses throughout the plant [158]. Inoculation of PGPR *Serrattia marcescens* (Bizio, 1823) [159] strain 90–166 and *P. fluorescens* WCS417r showed rapid upregulation of several JA-biosynthetic and -responsive genes, e.g., *VSP*, *PDF1.2*, *HEL*, *ChiB*, *LOX,* and *PAL* in systemic (leaf) tissue throughout a number of crop plants, as well as *Arabidopsis* [61,160]. JA production then coordinates with abscisic acid (ABA) signaling to activate selective stress-responsive transcription factors such as *OCP3* (*Overexpressor of Cationic Peroxidase 3*) and *MYB60* [161,162], which in turn regulate stomatal closure, and suppress plant growth and yield [163]. These signaling and metabolic cascades may explain the inhibitory effect of JA in plant growth and development [164]. Indeed, the exogenous application of JA characteristically results in the inhibition of root growth [6,165,166], together suggesting that (i) ISR (and PGPR-induced JA signaling) could compete resource allocations (namely, defense and growth tradeoffs) with PGPR-mediated growth enhancement, or (ii) ISR employs alternative and/or additional (besides JA-responsive) signaling and/or metabolic pathways to prime plant disease resistance without reducing yield potential.

### 3.3. Potential Roles of SA in the Indirect Antagonism, SAR of PGPR against PPN

Recently, selective data argues that PGPR could stimulate SAR (instead of ISR), widely known to be developed by pathogenic microbes but not by PGPR [7,131]. The caveat is that ISR and SAR require mutually antagonistic cellular mechanisms, JA signaling for ISR versus SA signaling for SAR [6,7], which initially conflicted the role of SAR and SA signaling in the indirect antagonism of PGPR. However, a few studies reported that several *Bacillus* spp. can stimulate SA accumulations and signaling in plants (Table 2), hypothesizing that a mode of ISR mimics or shares SAR mechanisms. For instance, *B. amyloliquefaciens* MBI600, active ingredient of the biological fungicide Serifel^®^, was characterized to activate SA-dependent, but JA-independent, immunity, and reduce the disease severity of tomato spotted wilt virus in tomatoes [61]. It is notable that independent studies from other groups using similar PGPR strains such as *B. amyloliquefaciens*, *B. mycoides* (Flugge, 1886) [167], *B. pumilus* (Meyer and Gottheil, 1901) [168], and *B. subtilis* reported an induction of JA signaling (see Table 2). The cause for this contradiction is not understood, but may result from different experimental conditions and/or experimental errors. Alternatively, there may be timing differences to induce JA or SA signaling by PGPR (JA first and SA latter). A recent study using time-resolved transcription analyses indicated that the inoculation of *Trichoderma harzianum* (Rifai, 1969) [169] rapidly activates JA signaling on a time scale of hours, but later switches to enhance SA activity in 3 to 7 days in both infected and systemic tissues of plants [89]. This finding suggests the presence of complex and/or alternative mechanisms in PGPR-mediated systemic resistance. Further investigations must clarify the combined or antagonistic roles and functions of different plant hormone signaling such as JA, ET, SA, and ABA [170] in conveying defense activation by PGPR.

### 3.4. Crosstalk between SA and JA Signaling

As alluded, JA and SA signaling crosstalk with each other [171,172], and these interactions can be either antagonistic or synergistic. One of the first investigations into their interaction revealed an antagonistic relationship between JA and SA in tomatoes. They observed that aspirin, formulated SA, significantly suppressed the expression of JA marker genes [173]. Similarly, the expression of SA marker genes was inhibited upon the stimulation of JA by *Pseudomonas syringae* in tomato [174]. Furthermore, the application of exogenous JA also correlated with decreased SA activity, and enhanced JA signaling [175]. A more in depth look into the antagonism demonstrated that SA signaling targets the downstream of promoter in JA-responsive genes, and inhibits any further JA activity [176]. It is hypothesized that the antagonism be-tween the two hormones (JA and SA) helps plants conserve energy, and optimize the defense pathways in the presence of a single pathogen [171,172].

In contrast, a few early studies suggested a potential, synergistic relationship between JA and SA, as the pathogens *Botrytis cinerea* (Persoon, 1794) [177] and Tobacco mosaic virus stimulate both JA and SA at the same time [178,179]. Mur et al. later suggested that the concentration of hormones determined the interaction between them [180]. If the level of JA or SA is too high there was an antagonistic relationship; however, if the levels of each hormone were lower, the two hormones acted synergistically [180]. The exact concentration where synergism between the two hormones turns to antagonism is yet to be determined.

The goal of biological control research is to optimize the BCA to be as effective and efficient as possible against the target pathogen(s), and determining any manipulation of the JA and SA pathways by the target pathogen is an essential part of this type of research. This can, in turn, determine the best pathway to provide the most effective protection and defense against the target pathogen. This is important to keep in mind when selecting the best BCA in each management strategy.

## 4. Commercialization of PGPR

There is still a fundamental question (i.e., reproducibility) to address for the commercialization of PGPR, though multiple strains of PGPR are available on the market as biological nematicides (Table 3). However, the efficacy of these products needs to be further evaluated. To be commercially successful, PGPR products should have a broad application, long shelf life, be safe to use, have a viable market, easy availability, be consistent and have a low investment cost. Indeed, a large number of PGPR strains still remain to be investigated (Table 4). One particularly noticeable genus of PGPR is *Bacillus* spp. (9 direct antagonism, 7 indirect antagonism, and 17 uncharacterized); considered to be good options because they can quickly replicate and colonize plants, tolerate harsher environments, and easily form endospores. Moreover, they are documented to affect a broad spectrum of plant pathogens including PPN, viruses, bacteria, and fungi [181]. An extra benefit is that many of these *Bacillus* spp. can promote plant growth and help the plant adapt abiotic stresses, enhancing yield potential [182,183]. *Bacillus* spp. are the main species of commercialized PGPR due to their hardiness, as described above, compared to other effective PGPR such as *Pseudomonas* spp. [184]. More studies however are required to assess more effective mechanisms of their modus operandi as BCAs of PPN.

## 5. Conclusions

Emerging results have proposed the potential use and value of PGPR as BCAs in managing PPN. Selective strains of PGPR, in particular many *Bacillus* spp. appear to effectively suppress the growth and infestation of PPN in plants and fields, via direct or indirect antagonistic mechanism. Initially, much effort was invested to isolate (1) predatory PGPR strains and/or (2) PGPR-released nematicidal toxins and hydrolytic enzymes, which readily kill or inhibit PPN. However, their products were often highly host-specific, limiting actual use and practicality for field applications. Thus, recent studies have tried to understand, and improve the indirect antagonism (ISR) of PGPR, especially since PGPR have (i) longer shelf life, (ii) easy availability, (iii) low investment- and production-cost, and are (iv) safe to use. Moreover, PGPR are (v) effective biostimulants facilitating plant growth and yield potential, as well as are able to (vi) enhance plant resistance against a broad range of microbial pathogens, pests, and insect herbivores (i.e., ISR), and (vii) concurrently prime plants to better adapt or acclimate towards various abiotic stresses including drought, salinity, and extreme temperatures. These multifaceted activities, and benefits clearly underpin important merits and practicality for further screening and accessing the activity and efficacy of new PGPR in managing economically damaging PPN. Successful development/commercialization of BCAs will greatly aid in upgrading plants’ own survival capacity against various ecological constraints, but without trading off yield potential.

## Figures and Tables

**Table 1:** A list of plant growth-promoting rhizobacteria (PGPR) conferring direct antagonisms against plant-parasitic nematodes (PPN).

PGPR	Target PPN	Molecules or Modes	References
*Bacillus cereus*	*Heterodera avenae,* *Meloidogyne incognita,* *Meloidogyne javanica*	Sphingosine, Protease, Chitinase, Antibiotic production, Secondary metabolites	Oka et al., 2008 [9]; Gao et al., 2016 [10]; Ahmed, 2019 [11]
*Bacillus coagulans*	*Meloidogyne incognita*	Hydrolytic enzymes	Ambo et al., 2010 [12]; Serfoji et al., 2013 [13]; Xiang et al., 2018 [4]
*Bacillus firmus*	*Ditylenchus dipasi, Heterodera* spp., *Meloidogyne incognita, Pratylenchus* spp., *Radopholus similis*	Sep 1 protease, Secondary metabolites	Giannakou et al., 2004 [14]; Mendoza et al., 2008 [15]; Terefe et al., 2009 [16]; Terefe et al., 2012 [17]; Xiong et al., 2015 [18]; Geng et al., 2016 [19]; Bayer Crop Science [20]
*Bacillus licheniformis*	*Bursaphelenchulus xylophi lus, Meloidogyne incognita*	Protease, Chitinase	Siddiqui and Husain 1991 [21]; Siddiqui and Mahmood 1992 [22]; Jeong et al., 2015 [23]; El-Nagdi et al., 2019 [24]
*Bacillus megaterium*	*Heterodera glycines,Meloidogyne incognita*, *Meloidogyne graminicola*	Protease, Secondary metabolites	Kloepper et al., 1992 [25]; Padgham et al., 2007 [26]; Mostafa et al., 2018 [27]
*Bacillus pumilus* L1	*Heterodera glycines*, *Meloidogyne arenaria*	Protease, Chitinase	Lee and Kim 2015 [28]; Forghani and Hajihassani et al., 2020 [29]
*Bacillus subtilis*	*Helicotylenchus multicinctus,* *Meloidogyne graminicola, Meloidogyne incognita, Meloidogyne javanica, Rotylenchulus reniformis*	Lipopeptide antibiotics, Hydrolytic enzymes, Secondary metabolites	Prakob et al., 2009 [30]; Kavitha et al., 2012 [31]; Basyony and Abo-Zaid, 2018 [32]; Mazzuchelli et al., 2020 [33]; Gautam et al., 1995 [34]
*Bacillus thuringiensis*	*Heterodera glycines, Meloidogyne incognita*	*Bt* crystal protein (toxin protein), Thuringiensin (β-exotoxin)	Noel, 1990 [35]; Wei et al., 2003 [36]; Mohammed et al., 2008 [37]
*Corynebacterium paurometabolum*	*Meloidogyne incognita*	Hydrogen sulfide, Chitinase	Mena and Pimentel, 2002 [38]
*Pasteuria penetrans* ^1^	*Meloidogyne* spp.	Predation	Mankau et al., 1976 [39]; Mankau and Prasad, 1977 [40]; Dube and Smart, [41]; Sayre and Starr 1975 [42]; Bhuiyan et al., 2018 [43]
*Pasteuria thornei* ^1^	*Pratylenchus* spp.	Predation	Mankau et al., 1976 [41]; Atibalentja et al., 2000 [44]
*Pasteuria nishizawae* ^1^	*Globodera* spp., *Heterodera* spp.	Predation	Sayre and Wergin, 1991 [45]
*Pseudomonas aeruginosa*	*Caenorhabditis elegans, * *Meloidogyne incognita, Meloidogyne javanica*	Hydrogen cyanide (HCN)	Siddiqui and Ehteshamul-Haque, 2001 [46]; Gallagher and Manoil, 2001 [47]; Singh and Siddiqui, 2010 [48]
*Pseudomonas. fluorescens* F113	*Globodera rostochinensis*	2,4-diacetylphloroglucinol (DAPG)	Cronin et al., 1997 [49]
*Pseudomonas fluorescens* CHA0	*Meloidogyne incognita*, *Meloidogynejavanica*	HCN, DAPG, Pyoluteorin, Extracellular protease	Siddiqui and Shaukat, 2003 [50]; Hamid et al., 2003 [51]; Siddiqui et al., 2005 [52]
*Pseudomonas fluorescens* Wood1R	*Meloidogyne incognita*	DAPG	Timper et al., 2009 [53]
*Pseudomonas stutzeri*	*Meloidogyne incognita*	HCN	Khan et al., 2016 [54]
*Serratia marcescens*	*Meloidogyne incognita,* *Meloidogyne javanica,* *Radopholus similis*	Volatile metabolites, Prodigiosin	Zabaketa-Mejia, 1985 [55]; Rahul et al., 2014 [56]

^1^ nematode parasitic bacteria.

**Table 2:** A list of PGPR conferring indirect antagonisms against PPN.

PGPR	Target PPN	Modes	References
*Agrobacterium radiobacter* (G12)	*Globodera* spp.	ISR	Hasky-Guenther et al., 1998 [57]; Hackenberg and Sikora, 1992 [58]; Racke and Sikora, 1992 [59]; Hackenberg et al., 1999 [60]
*Bacillus amyloliquefaciens* (syn. *Bacillus velezensis*)	*Heterodera glycine,* *Meloidogyne incognita*	ISR and SAR	Ryu et al., 2004 [61]; Beris et al., 2018 [62]; Burkett- Cadena et al., 2008 [63]; Choudhary et al., 2009 [64]; Li et al., 2015 [65]; Xiang et al., 2016 [66]; Xie et al., 2018 [67]
*Bacillus cereus*	*Meloidogyne javanica, Meloidogyne incognita*	ISR	Xiang et al., 2016 [66]; Halfeld-Vieira et al., 2006 [68]; Niu et al., 2011 [69]; Jiang et al., 2020 [70]
*Bacillus mojavensis*	*Meloidogyne incognita*	ISR	Xiang et al., 2016 [66]; Liu et al., 2016 [71]
*Bacillus mycoides*	*Meloidogyne incognita*	ISR and SAR	Xiang et al., 2016 [66]; Barbagus et al., 2004 [72]
*Bacillus pasteurii*	*Meloidogyne incognita*	ISR	Xiang et al., 2016 [66]; Ryu et al., 2003 [73]
*Bacillus pumilus*	*Heterodera glycine,* *Meloidogyne incognita*	ISR and SAR	Xiang et al., 2016 [66]; Zhang et al., 2002 [74]; Barbagus et al., 2004 [75]; Kavitha et al., 2007 [76]; Choudhary et al., 2007 [77]; Lastochkina et al., 2017 [78]
*Bacillus sphaericus*	*Globodera pallida, Meloidogyne incognita*	ISR	Hasky-Guenther et al., 1998 [57]; Racke and Sikora 1992 [59]; Xiang et al., 2016 [66]
*Bacillus subtilis*	*Heterodera cajani, Meloidogyne arenaria, Meloidogyne incognita, Meloidogyne javanica*	ISR and SAR	Ryu et al., 2004 [61]; Xiang et al., 2016 [66]; Kavitha et al., 2007 [76]; Choudhary et al., 2007 [77]; Lastochkina et al., 2017 [78]
*Bacillus thuringiensis*	*Aphelenchus avenae, Meloidogyne incognita*	ISR	Zhang et al., 2002 [74] Akram et al., 2013 [79]; Zuckerman et al.; 1993 [80]
*Pseudomonas aeruginosa*	*Meloidogyne javanica*	ISR and SAR	Audenaert et al.,2013 [81]; Fatima et al., 2017 [82]
*Pseudomonas fluorescens*	*Meloidogyne incognita, Meloidogyne javanica*	ISR and SAR	Siddiqui and Shaukat 2003 [50]; Choudhary et al., 2007 [77]; Krechel et al., 2002 [83]; Saikia et al., 2013 [84]; de Vleesschauwer et al., 2012 [85]; Leeman et al., 1995 [86]
*Pseudomonas putida*	*Meloidogyne incognita*	ISR	Krechel et al., 2002 [83]; Almaghrabi et al., 2013 [87]
*Rhizobium etli*	*Meloidogyne* spp.	ISR	Reitz et al., 2000 [88]
*Serratia marcescens*	*Meloidogyne incognita*	ISR	Zhang et al., 2002 [74]; Almaghrabi et al., 2013 [87]
*Trichoderma harzianum* ^1^	*Meloidogyne incognita*	ISR and SAR	Martínez-Medina et al., 2017 [89]

^1^ plant growth-promoting fungi (PGPF).

**Table 3:** A list of PGPR commercialized as BCAs against PPN.

Commercial Products	PGPR	Applications	References
BioNemaGon^TM^	*Bacillus firmus*	Reduce nematode population and root infestation by nematodes in vegetables and herbs	[90]
BioYield^TM^	*Bacillus subtilis GB03, Bacillus amyloliquefaciens*	Nematodes in tomato, strawberry, and bell pepper	[65]
Clariva^®^ pn	*Pasteuria nishizawae Pn1*	Seed treatment; Target *Heterodera glycines* to reduce feeding and reproduction, and increase yields under heavy PPN pressure.	[91]
Deny, Blue Circle	*Burlkholderia cepacia*	Inhibit egg hatching and mobility of nematode juveniles	[92]
MeloCon^®^, BioAct and NemOut	*Purpureocillium lilacinus* 251	Inhibit root knot, burring, cyst, reniform, spiral, sting, and root lesion nematodes.	[93]
Naviva ST	*Pasteuria sp. Ph3*	Seed treatment; Inhibit *Rotylenchulus reniformis* in cotton, soy, vegetables, cucurbits, and floriculture.	[94]
NewPro	*Pasteuria usgae* Bl1 + *Pasteuria* sp. Ph3	Inhibit lance and sting nematodes in turf (Bermudagrass and St. Augustine grass)	[95]
Nortica 10 WP	*Bacillus firmus* I-1582	Inhibit cyst, lance, lesion, ring, root knot sheath, spiral, sting, and stunt nematodes in turf.	[96]
VOTiVO FS	*Bacillus firmus* I-1582	Seed treatment; inhibit a broad range of nematodes. Available also as premix with insecticide	[97]

**Table 4:** A list of PPN inhibitory PGPR with uncharacterized function.

PGPR	Target PPN	Target Crops	References
*Alcaligenes faecalis*	*Meloidogyne incognita*	Chickpea	Siddiqui and Mahmood, 1992 [22]
*Azotobacter chroococcum*	*Meloidogyne incognita, Meloidogyne javanica*	Eggplant, Tomato	Bansal et al., 2002 [98]
*Bacillus altitudinis,* *Bacillus aerophilus,* *Bacillus aryabhattai,* *Bacillus galliciensis,* *Bacillus psychrosaccharolyticus*, *Bacillus safensis,* *Bacillus siamensis,* *Bacillus simplex,* *Bacillus toyonensis,* *Bacillus weihenstephanensis*	*Meloidogyne incognita*	Cotton	Xiang et al., 2016 [66]
*Bacillus firmus*	*Belonolaimus longicaudatus*	Bermudagrass	Crow, 2014 [99]
*Bacillus flexus*	*Meloidogyne incognita*	Basil	Tiwari et al., 2017 [100]
*Bacillus isolates*	*Heterodera cajani, Meloidogyne incognita*	Pigeon pea	Siddiqui and Shakeel, 2007 [101]
*Bacillus methylotrophicus*	*Meloidogyne incognita*	Tomato	Zhou et al., 2016 [102]
*Bacillus polymyxa*	*Meloidogyne incognita*	Tomato	Khan and Akram, 2000 [103]
*Bacillus tequilensis*	*Meloidogyne incognita*	Basil	Tiwari et al., 2017 [100]
*Burkholderia cepacia*	*Meloidogyne incognita*	Tomato	Meyer et al., 2000 [104]
*Lysinibacillus sphaericus*	*Meloidogyne incognita*	Tomato	Colagiero et al., 2018 [105]
*Lysobacter* spp.	*Meloidogyne incognita*	Tomato	Zhou et al., 2016 [102]
*Paenibacillus lentimorbus, Paenibacillus polymyxa*	*Meloidogyne incognita*	Tomato	Son et al., 2009 [106]
*Paenibacillus macerans*	*Meloidogyne exigua*	Coffee	Oliveira et al., 2007 [107]
*Pseudomonas solanacearum*	*Rotylenchulus reniformis*	Eggplant	Kermarrec et al.,1994 [108]
*Pseudomonas striata*	*Meloidogyne incognita*	Pea	Siddiqui and Singh, 2005 [109]
*Pseudomonas stutzeri*	*Meloidogyne incognita*	Chickpea	Seenivasan et al., 2001 [110]
*Stenotrophomonas maltophilia*	*Paratrichodorus pachydermus, Trichodorus primitivus*	Potato	Insunza et al., 2002 [111]
*Streptomyces* spp.	*Meloidogyne incognita*	Eggplant, Tomato	Rashad et al., 2015 [112]
